# *Sargassum horneri* (Turner) C. Agardh ethanol extract inhibits the fine dust inflammation response via activating Nrf2/HO-1 signaling in RAW 264.7 cells

**DOI:** 10.1186/s12906-018-2314-6

**Published:** 2018-09-10

**Authors:** Thilina U. Jayawardena, K. K. Asanka Sanjeewa, I. P. Shanura Fernando, Bo Mi Ryu, Min-Cheol Kang, Youngheun Jee, Won Woo Lee, You-Jin Jeon

**Affiliations:** 1 Department of Marine Life Sciences, Jeju National University, Jeju, 690-756 Republic of Korea; 2 Department of Veterinary Medicine and Veterinary Medical Research Institute, Jeju National University, Jeju, 690-756 Republic of Korea

**Keywords:** *Sargassum horneri* (Turner) C. Agardh, Inflammation, Oxidative stress, RAW 264.7 macrophages, Nrf2/HO-1, p38 MAPK

## Abstract

**Background:**

Among the different kinds of pollution, air pollution continues to increase globally. East Asia is considered to be significantly affected. As a result, the populations in these regions face serious health issues including respiratory disorders. This study investigated the impact of fine dust (FD) particles (CRM No. 28) on macrophage cells as a model for alveolar lung cells.

**Methods:**

The research focused on inflammation and oxidative stress induced by FD and *Sargassum horneri* (Turner) C. Agardh ethanol extract (SHE) as a potential treatment. *S. horneri* is a type of brown algae that has demonstrated anti-inflammatory effects against RAW 264.7 macrophages in previous studies. MTT, Griess, ELISA, western blotting, and mRNA expression analyses using PCR techniques were used in this study.

**Results:**

The optimum FD concentration was determined to be 125 μg mL^− 1^. FD particles stimulated inflammatory mediators production (iNOS, COX-2, and PGE_2_) and pro-inflammatory cytokines (IL-1β, IL-6, and TNF-α), leading to NO production. These mediators were dose-dependently downregulated by treatment with SHE. IL-6 and TNF-α were identified as biomarkers for FD. SHE treatment induced HO-1 and Nrf2 activity in a dose-dependent manner under FD stimulation. This confirmed the cytoprotective effect against oxidative stress induced via FD. Furthermore, treatment of the cells with a p38 MAPK inhibitor (SB202190) induced FD-stimulated NO production.

**Conclusions:**

The results suggest that SHE increases macrophage cellular resistance to FD-induced inflammation and oxidative stress, probably via the p38 MAPK pathway and Nrf2/HO-1 expression.

## Background

Humans and other organisms face disastrous situations every day. As we are currently living in the fourth industrial revolution, each country is moving towards fast-paced development. More pollutants are being released into the natural environment. Industrial zones and traffic emit volatile hydrocarbons, airborne particles, nitrogen oxides, and carbon monoxide. These particles are a complex mixture of both inorganic and organic matter consisting of varying mass, size, and chemical composition [1]. This has become a major concern of increasing interest globally and particularly in the East Asian region, including China, Korea, and Japan. These fine dust particles could have detrimental effects on humans and other organisms. A major natural contributor is the extensive arid or semiarid highlands of northern China and Mongolia (i.e., Taklimakan desert, Gobi desert, Loess Plateau, and Hunshdak Sandy Lands). It was recently reported by Lee et al. (2015) that compared to this natural source, emissions released by industry in China are much smaller [2].

These particles of fine dust (FD) are reported to have an aerodynamic diameter equal to or less than 10 μm (PM10) [1]. Particles of this size could easily penetrate the respiratory tract. Human health is affected by these FD particles, and continued exposure could result in detrimental issues including complications in the respiratory tract, allergic reactions, and inflammation responses in macrophage cells. As the human body consists of natural defense mechanisms, alveolar macrophages mark the first line of defense. Alveolar macrophages are involved in phagocytosis of inhaled particles. As a model system in our study, the RAW 264.7 cell line was used. Recently, many studies have been conducted regarding the activity of RAW 264.7 macrophage inflammatory responses against FD stimulation. Cytokine release (TNFα, IL-6, and IL-1β) and expression of genes/proteins like iNOS and COX-2 are characteristic features of promoting inflammatory responses [3]. Zhao et al. (2016) reported that FD induced an inflammatory response through the ROS-dependent pathway [4]. Inside the lungs, microbe elimination is supported by ROS, but the production of ROS results in oxidative stress. Hence, the lung cell components are damaged, which leads to respiratory disorder. Excessive inflammation and oxidative stress are reported as the main underlying events in respiratory diseases [5, 6].

*S. horneri* is a well-known edible seaweed [7] that is widely used in the Asia-Pacific region. It has been reported to possess numerous immunological activities anti-inflammatory potential. Different single components have been isolated from the species, and these extracts are also reported to have good biological activities. Kim et al. (2014) reported the potential anti-inflammatory activity of the ethanol extract of *S. horneri* [8]*.* However, to the best of our knowledge, this is the first study regarding *S. horneri* ethanol extract (SHE) activity against FD-induced macrophage cells.

This research was performed to identify the effects of SHE on the regulation of inflammatory responses in macrophages stimulated by FD particles. We also further examine the mechanism by which SHE could possibly regulate and modulate inflammatory responses. The focus was on the oxidative stress caused by inflammation responses. The effects of SHE treatment on p38 and inhibiting the MAPK and Nrf2/HO-1 pathways were investigated.

## Methods

### Materials

CRM certified FD reference material (CRM No. 28 Urban Aerosols) was purchased from the Center for Environmental Measurement and Analysis, National Institute for Environmental studies, Ibaraki, Japan. 3-(4,5-dimethylthiazol-2-yl)-2,5-diphenyltetrazolium bromide (MTT) was purchased from Sigma-Aldrich (St. Louis, MO, USA). All analytical grade organic solvents were purchased from Sigma-Aldrich. RAW 264.7 macrophage cells were purchased from Korean Cell Line Bank (KCLB, Seoul, Korea). Growth medium used in the experiment, Dulbecco’s modified Eagle’s medium (DMEM), and antibiotics (penicillin and streptomycin) were purchased from GIBCO Inc. (Grand Island, NY, USA). Antibodies used in western blot analysis were purchased from Santa Cruz Biotechnology (Santa Cruz, CA, USA). All the cytokine kits used during the experiment were purchased from eBioscience (San Diego, CA, USA), R&D Systems (Minneapolis, MN, USA), BD Opteia (San Diego, CA, USA), and Invitrogen (Carlsbad, CA, USA).

### Extraction from *Sargassum horneri*

*S. horneri* samples were collected from the coast of Jeju Island, South Korea, in January 2017. The sample identification was assisted by Dr. Ki-Wan Lee. Sample repositories (SH2017J005) were kept in the Laboratory of Marine Bioresource Technology at Jeju National University. The ethanol extraction procedure followed the previously published method [9]. Briefly, with the intention of removing salt, sand, and epiphytes, collected seaweed was washed with running water. Samples were then lyophilized and ground into fine powder. A sample powder (50 g) was extracted into 70% ethanol (10% *w*/*v*) with the assistance of a shaking incubator. This was centrifuged and filtered, and the filtrate was lyophilized, resulting in the ethanol extract (SHE) used in subsequent experiments. The percent yield of SHE was 1.44%. The SHE was dissolved in dimethyl sulfoxide (DMSO) to prepare a stock solution and was diluted using PBS in order to prepare working concentrations. Hence, we managed to maintain the final concentrations of DMSO in treated samples less than 0.1% [10, 11].

### Fine dust particle size estimation using SEM

A Q150R rotary-pumped sputter coater (Quorum Technologies, Lewes, UK) was utilized for the FD specimen sputter-coating procedure with platinum. Using a JSM-6700F field-emission scanning electron microscope (JEOL, Tokyo, Japan), the surface morphology of CRM No. 28 particles was observed. The instrument was operated at 10.0 kV [12].

### Cell line maintenance

DMEM supplemented with 10% FBS and 1% antibiotics was used to maintain the RAW 264.7 macrophages. Cells were maintained in a humidified atmosphere with a 5% CO_2_ level and 37 °C temperature. Cells were periodically subcultured, and the cells were exposed to the experimental conditions during the exponential growth phase.

### Cell viability measurement

Evaluation of cytotoxicity against FD and FD-induced macrophages with SHE was performed using the MTT assay [13]. The cells were seeded in 24-well plates at a cell concentration of 1 × 10^5^ cells mL^− 1^ and treated with the relevant sample (FD and FD-induced SHE treated) after a 24 h incubation period. FD was suspended in DMEM and homogenized achieving a final concentration of 2.5 mg mL^− 1^, and serial dilution was performed. The final concentrations were 15.6, 31.3, 62.5, 125, and 250 μg mL^− 1^ and 50 μg mL^− 1^ LPS. The MTT assay was performed, and the optical density was measured at 540 nm. Based on the results, the concentration of FD used for subsequent cell experiments was identified.

### NO inhibition activity evaluation

RAW 264.7 macrophages were seeded in 24-well plates (1 × 10^5^ cells mL^− 1^). Following a 24 h incubation period, the wells were treated with SHE at different concentrations. After 1 h incubation, the wells were stimulated using FD (125 μg mL^− 1^) and further incubated for 24 h. NO production and cell viability were measured by Griess and MTT assays [14]. The level of NO inhibition was evaluated as the ratio of the mean percentages of NO production by cells exposed to LPS to FD.

### Assessing PGE_2_ and pro-inflammatory cytokine production

Cell culture and seeding experiments were performed in a similar manner as described above. Culture media was collected from each well to determine the expression of prostaglandin E2 (PGE_2_), tumor necrosis factor α (TNF-α), interleukin (IL)-1β, and IL-6. Each immunoassay was performed in accordance with the manufacturer instructions.

### Western blot analysis

At 24 h after sample treatment and subsequent FD stimulation, RAW 264.7 cells were harvested. Proteins were collected from the harvested cell lysates [15]. The protein levels were evaluated using the BCA protein assay kit (Bio-Rad, USA). Sodium dodecyl sulfate-polyacrylamide gels (12%) were used for the electrophoresis. Subsequently, the transfer was completed on nitrocellulose membranes. The membranes were incubated overnight with specific primary antibodies β-actin, HO-1, Nrf-2, iNOS, and COX-2 (Santa Cruz Biotechnology) in 5% skim milk. After the incubation, the HRP-conjugated secondary antibodies (anti-mouse IgG, Santa Cruz Biotechnology) were added to the membrane. Then, signals were developed via the addition of chemiluminescent substrate (Cyanagen Srl, Bologna, Italy). Membranes were photographed using a FUSION SOLO Vilber Lourmat system. The band intensities were quantified using the ImageJ program [16].

### RNA extraction and cDNA synthesis

Using the Tri-Reagent™ extraction kit (Sigma-Aldrich, St. Louis, MO, USA) and following the given instructions, the total RNA from RAW 264.7 cells was extracted. The purity of the extracted RNA was evaluated using the μDrop Plate (Thermo Scientific). Next, RNA samples were diluted (1 μg μL^− 1^). For the purpose of synthesizing first-strand cDNA, another kit was used (prime Script™) (TaKaRa BIO INC, Japan) following the manufacturer instructions. Synthesized cDNA was stored at − 80 °C.

### Quantitative real-time PCR (qPCR) analysis

Using GAPDH as an internal reference standard gene in the amplification process, the pro-inflammatory cytokine expression levels were evaluated using SYBR Green quantitative real-time PCR (qPCR). This was performed using a Thermal Cycler Dice-Real Time System (TaKaRa, Japan). The primers used in this experiment are described in Table 1 (Bioneer, Seoul, Korea).

**Table 1:** Sequence of the primers used in this study

Gene	Primer	Sequence
GAPDH	Sense	5′- AAGGGTCATCATCTCTGCCC-3′
	Antisense	5′-GTGATGGCATGGACTGTGGT-3′
iNOS	Sense	5′-ATGTCCGAAGCAAACATCAC-3′
	Antisense	5′-TAATGTCCAGGAAGTAGGTG-3′
COX-2	Sense	5′-CAGCAAATCCTTGCTGTTCC-3′
	Antisense	5′-TGGGCAAAGAATGCAAACATC-3′
IL-1β	Sense	5′-CAGGATGAGGACATGAGCACC-3′
	Antisense	5′-CTCTGCAGACTCAAACTCCAC-3′
IL-6	Sense	5′-GTACTCCAGAAGACCAGAGG-3′
	Antisense	5′-TGCTGGTGACAACCACGGCC-3′
TNF-α	Sense	5′-TTGACCTCAGCGCTGAGTTG-3′
	Antisense	5′-CCTGTAGCCCACGTCGTAGC-3′

A total of 10 μL reaction mixture was prepared using diluted cDNA (3 μL); gene specific forward and reverse primers (each 0.4 μL of 10 pM); ddH_2_O (1.2 μL); 2× TaKaRa ExTaq™, and SYBR premix (5 μL).

A thermal profile was used to perform the reaction as follows: Stage 1: 10 s at 95 °C; Stage 2: 40 cycles of 5 s at 95 °C, Stage 3: 10 s at 55 °C; Stage 4: 20 s at 72 °C; Stage 5: 15 s at 95 °C; Stage 6: 30 s at 55 °C; and Stage 7: 15 s at 95 °C. The relative expression levels were analyzed following the method described by Livak and Schmittgen (2001) [17].

### Anti-oxidative potential of SHE evaluation

The antioxidative potential of SHE against the FD-induced oxidative damage in RAW 264.7 cells was evaluated. Cytotoxicity and NO production were examined in the presence of MAPK inhibitors. The cells were treated with a selected concentration of SHE (62.5 μg mL^− 1^) for 24 h in the presence or absence of each selective inhibitor (SB 202190 = p38 inhibitor, SP 600125 = JNK inhibitor, and PD98059 = ERK inhibitor) or FD.

### Statistical analysis

All the data are expressed as the mean ± standard deviation of a minimum of three samples. A statistical comparison of significant differences was performed via IBM SPSS statistics using one-way ANOVA. *p* values less than 0.05 (*p* < 0.05) were considered significant.

## Results

### Fine dust composition

**Fig. 1:**
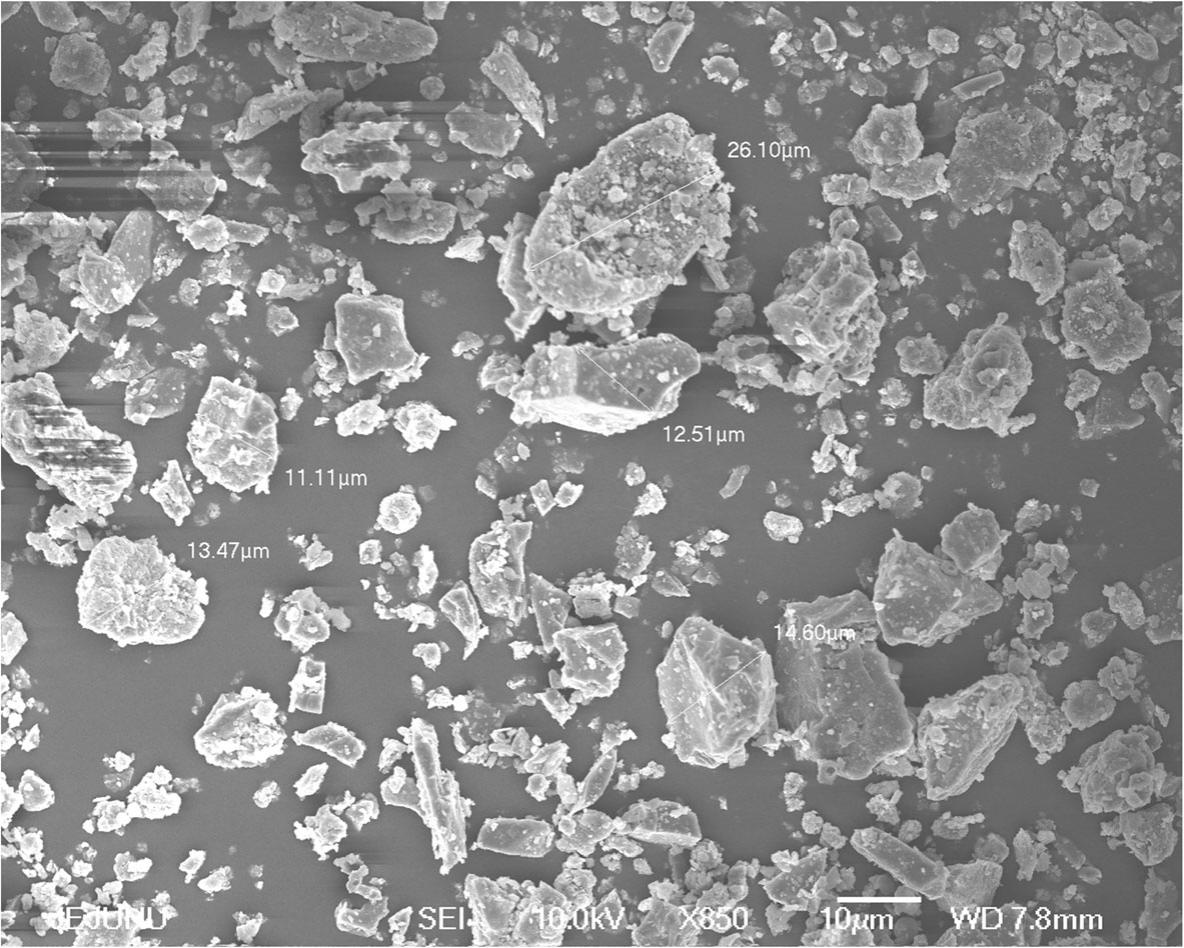
Scanning electron microscope image of fine dust (FD) particles. CRM No. 28 Urban Aerosols, purchased from the National Institute for Environmental studies, Ibaraki, Japan. Scale bar: 10 μm

Figure 1 shows the detailed image of the particles of CRM No. 28 Urban Aerosols, taken with a scanning electron microscope. Inconsistency in morphology throughout the sample was observed. CRM No. 28 is composed of particles of 2–14 μm in diameter. However, a large proportion of the particles had diameters < 2 μm. CRM No. 28 was certified to contain eight polycyclic aromatic hydrocarbons. Between them, benzo (b) fluoranthene has the highest mass fraction. Inorganic components, Mg, Ca, Ba and Sr, are reported as earth metals, while Mn and Pb are transition metal ions that occupy a much larger mass fraction in the FD sample.

### FD-induced inflammation in RAW 264.7 machrophages and anti-inflammatory potential of SHE

**Fig. 2:**
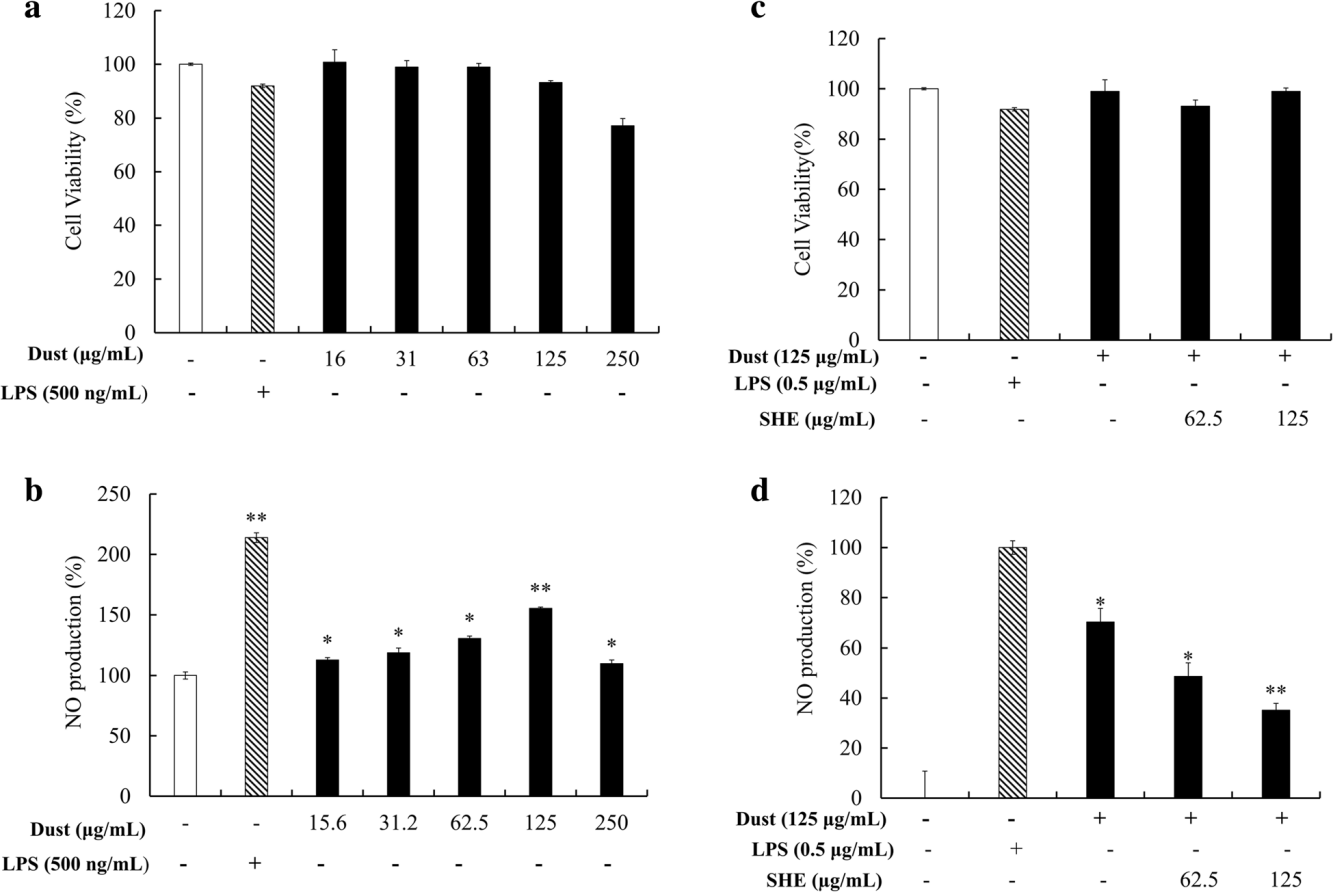
(**a**) Cytotoxicity and (**b**) NO production levels in FD-exposed RAW 264.7 cells. (**c**) Cytoprotective and (**d**) NO inhibitory effect of SHE in FD-exposed RAW 264.7 cells. RAW 264.7 cells seeded after 24 h treatment with SHE (62.5–125 μg mL^− 1^), incubation for 1 h, and co-treatment with culture medium and LPS (0.5 μg mL^− 1^) or FD (15.6–250 μg mL^− 1^). Triplicate experiments were used to evaluate the data, and the mean value is expressed as ± SD. *p < 0.05, **p < 0.01, in comparison to the respective control (ANOVA, Duncan’s multiple range test)

Figure 2a shows the stimulation of macrophages by both the LPS and FD particles. The stimulation increased NO production, and LPS induced an increased level compared to FD particles. For FD particles, a gradual increase was observed until 125 μg mL^− 1^, and there was a sudden decline in the 250 μg mL^− 1^ treatment group. The cytotoxicity experiment (Fig. 2b) also showed a sudden decrease in cell viability at the maximum level of treatment with FD particles. Consequently, the optimum FD stimulation concentration was evaluated to be 125 μg mL^− 1^.

Treatment with SHE (Fig. 2c, d) effectively and dose-dependently suppressed the NO production in FD-induced macrophages. Simultaneously, an increase in macrophage cell viability was also observed, which began at the SHE treatment concentration of 62.5 and continued up to 125 μg mL^− 1^.

### Regulation of anti-inflammatory mediators in FD-induced machrophages

**Fig. 3:**
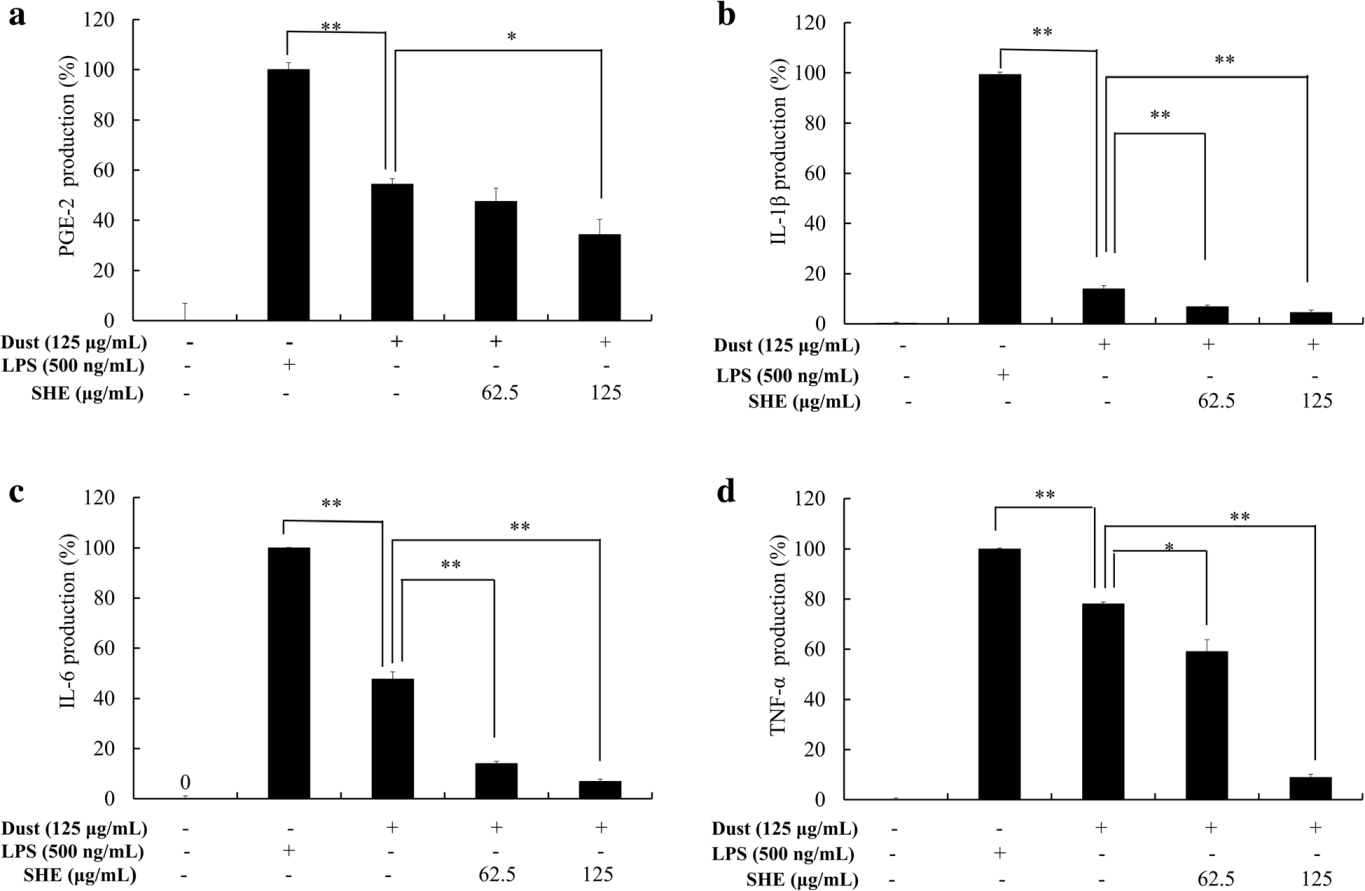
Inhibitory effect of SHE on the PGE2 and pro-inflammatory cytokines (IL-1β, IL-6, and TNF-α) production in FD-induced RAW 264.7 cells determined using ELISA. Culture supernatants of RAW 264.7 cells after successive treatment with LPS or FD were used to quantify the inflammatory cytokines and PGE2. Triplicate experiments were used to evaluate the data, and the mean value is expressed as ± SD. t-test was used to calculate the level of significance. * = *p* < 0.05 and ** = *p* < 0.01

**Fig. 4:**
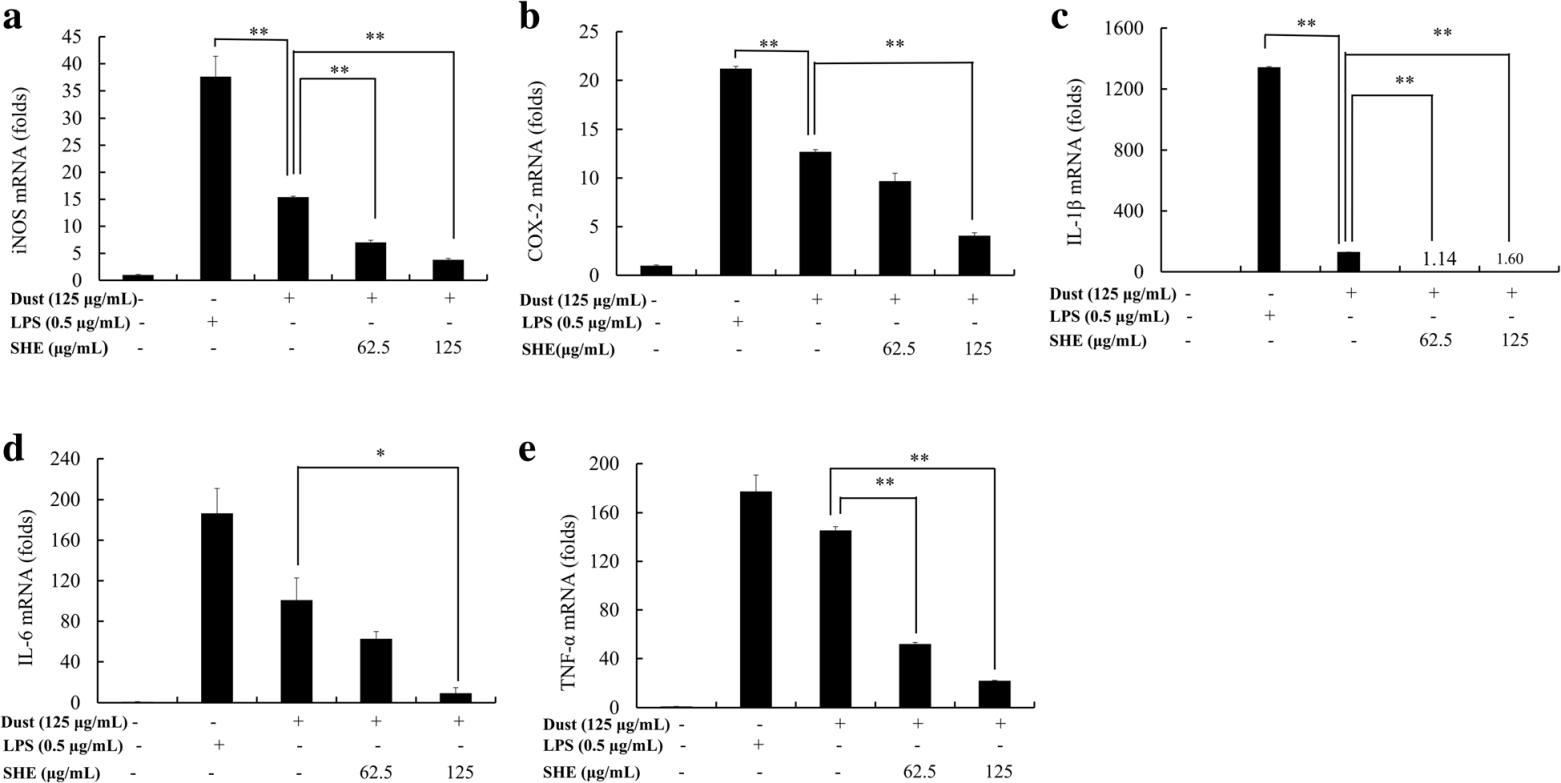
FD stimulated cells’ inflammation-associated gene expressions levels: (**a**) iNOS, (**b**) COX-2, (**c**) IL-1β, (**d**) IL-6, and (**e**) TNF-α. The 2^-ΔΔCt^ method was used to calculate the relative mRNA levels. GAPDH used as an internal reference. Triplicate experiments were performed. mRNA significance relative to the non-treated control was calculated using the Mann-Whitney U test. * = *p* < 0.05 and ** = *p* < 0.01

Western blotting showed (Fig. 3a, b) a change in nitric oxide synthase (iNOS) and cycloxyganse-2 (COX-2) protein levels in the LPS and FD stimulation groups, and FD-stimulated expression levels were lower than those of LPS. Treatment with SHE dose-dependently downregulated the levels of iNOS and COX-2, demonstrating the inflammatory inhibition activity of SHE. These are major inflammatory mediators that regulate the production of PGE_2_. It is evident that PGE_2_ is also regulated (Fig. 4a), coinciding with the previously observed results. Moreover, SHE treatment (Fig. 4b, c, d) significantly downregulated the FD-stimulated macrophage expression of IL-1β and IL-6, and TNF-α downregulation was notable but not significant. These results indicate that pre-treatment with SHE could suppress elevated pro-inflammatory cytokines, which are released into the media as a result of stimulation with FD. In addition, IL-6 and TNF-α demonstrated a sudden and significant increase as a result of FD stimuli compared to the LPS group.

Nrf-2 and HO-1 were expressed (Fig. 3c, d) initially as a result of the FD-stimulus, and a dose-dependent increase was observed with the co treatment with SHE. The change in nuclear Nrf2/HO-1 levels enhanced the production of cytoprotective factors such as superoxide dismutase (SOD).

### Effect of SHE on FD-induced macrophage gene expression

**Fig. 5:**
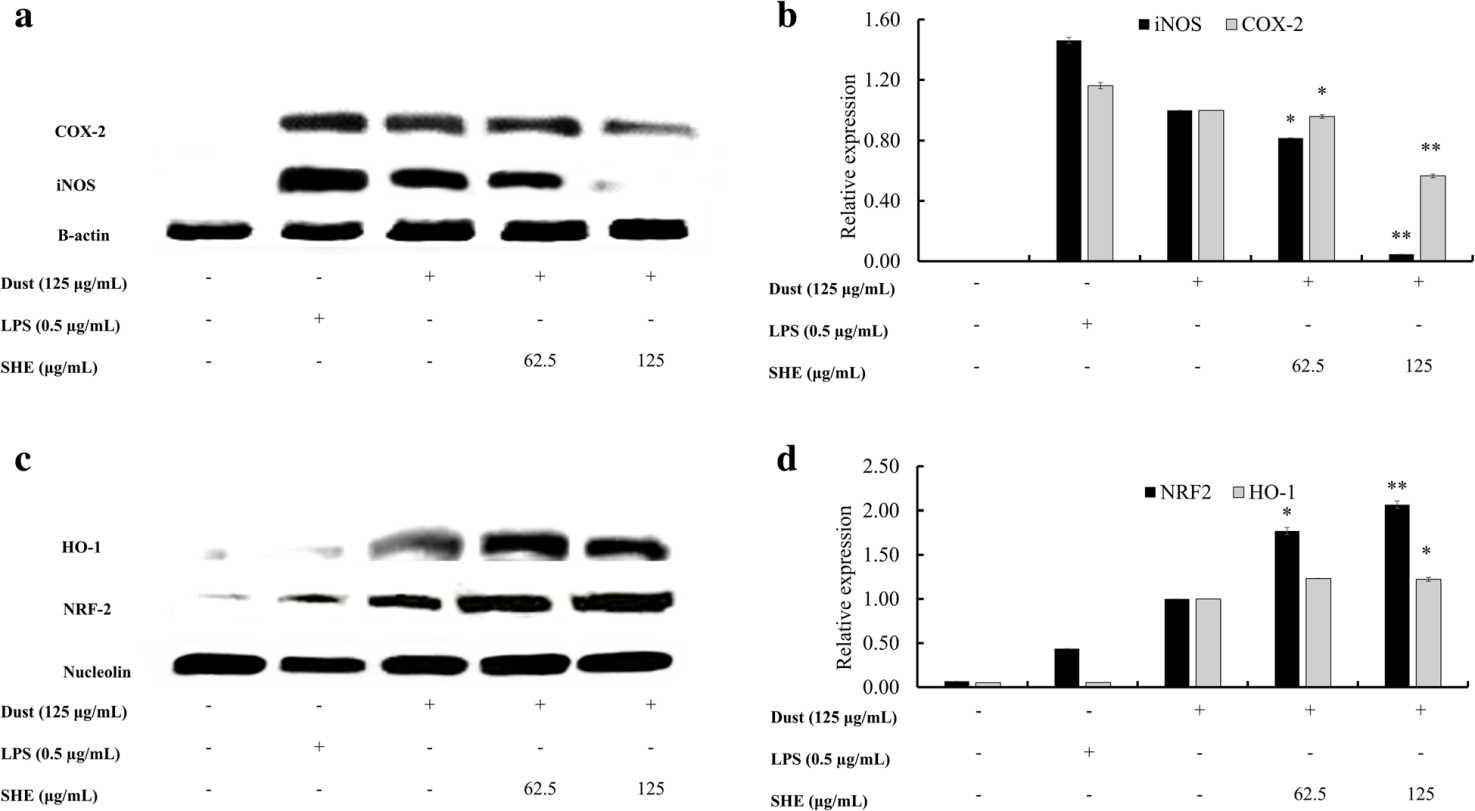
Inhibitory effects of SHE on FD-induced inflammation-associated protein in RAW264.7 cells. (**a**) iNOS and COX-2 levels were determined using western blotting. (**b**) Quantitative data. (**c**) SHE-induced HO-1 and Nrf2 measured by western blot, and (**d**) relevant quantitative data. β-actin (for the cytoplasm) and nucleolin (for the nucleus) were used as internal controls. Quantitative data was analyzed using ImageJ software. Results are expressed as the mean ± SD of three separate experiments. * = *p* < 0.05 and ** = *p* < 0.01

The qPCR results (Fig. 5a-e) showed a similar trend compared to the previous western blot results. mRNA levels of iNOS and COX-2 were decreased as a result of treatment with SHE in the FD-induced macrophage cells. Similarly, the mRNA expression levels of pro-inflammatory cytokines IL-6, IL-1β, and TNF-α were also reduced. These results further verify the potential of SHE to act as an anti-inflammatory agent.

### Fine dust-induced oxidative damage

**Fig. 6:**
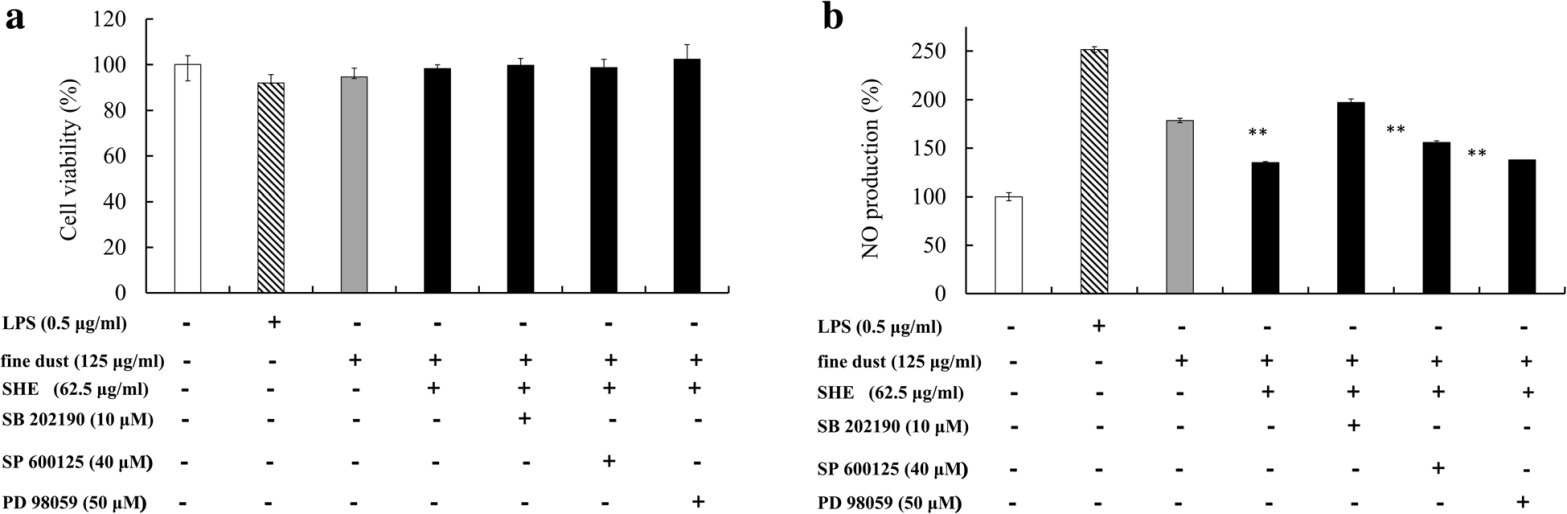
The antioxidative potential of SHE against the FD-induced oxidative damage in RAW 264.7 cells. (**a**) Cell viability and (**b**) NO inhibitory effect in the presence of specific MAPK inhibitors. The cells were treated with indicated concentrations of SHE (62.5 μg mL^− 1^) for 24 h in the presence or absence of each selective inhibitor. SB 202190 = p38 inhibitor, SP 600125 = JNK inhibitor, and PD98059 = ERK inhibitor. Triplicate experiments were used to evaluate the data, and the mean value is expressed as ± SD. Student’s t-test used to calculate the statistical significance. * = *p* < 0.05 and ** = *p* < 0.01

Figure 6 shows the stimulation of macrophages by FD and co-treatment with a selected SHE concentration. According to the results, LPS and FD-treated cells exhibited increased production of NO, but the effect from FD induction was less than that of LPS stimulation. Showing similarity with previous results, co-treatment with FD and SHE reduced NO production, while introducing SB 202190, the p38 inhibitor, upregulated the NO production significantly with a minimal effect on cell viability. Even though slight upregulation was observed with the SP600125 and PD98059 treatments, they did not exhibit a significant effect compared to FD and SHE co-treatment. These inhibitors were used to address the role of MAPK [18] in HO-1 expression by SHE and indirect expression via NO production.

## Discussion

In this study, we focused on both the inflammatory and cytotoxicity effects caused by exposure to outdoor air particles and also suggested a green chemical extract as a treatment.

Because of the fast pace of global industrialization, FD particles have become a major cause of air pollution. Countries in the Asia-Pacific region are faced with this issue, especially eastern China, Korea, and Japan. This is the result of large contributions of coal-burning power plants, vehicular traffic, heavy mining operations, and also naturally occurring sand storms. Different studies have been carried out in different parts of the world regarding FD chemical composition and its impact on human and animal behavior. Only few studies have been conducted focusing on the Asia-Pacific region. Among them, Choi et al. (2001) focused on the chemical signature of the FD of spring aerosol in Seoul, South Korea. He concluded that the Asian desserts are a dust source and also inferred that the chemical composition of FD is closely linked with air-mass trajectory [19]. Similarly, Maxwell et al. (2004) characterized Asian dust by its inorganic composition and reported that the main components of water-soluble mineral dust are Mg^2+^ and Ca^2+^. Additionally, most of the fine-particle negative ions remain as nitrate (NO_3_^−^) and sulfate (SO_4_^2−^) associated with ammonium (NH_4_^+^) or potassium (K^+^) [20]. In the present study, the physical parameters of the FD particles were evaluated, and the chemical parameters were obtained from the ERM certificate. Hence, the results show consistency with previous studies.

Among numerous biological mechanisms, inflammation has gained much attention among the scientific community because of its ability to mediate particulate-associated respiratory effects such as in asthma and pulmonary disease. Moreover, cardiovascular effects such as atherosclerosis and increased blood coagulation are also key parts of inflammation [21]. Bekki et al. (2016) reported that PM 2.5, a smaller dimension particle fraction of FD, can contribute to many adverse health effects. This particle size could easily pass through the throat and nose and reach the alveolus, and this study evaluated the effect of FD on lung alveoli using macrophage cells [22]. Macrophages inside the lungs act as a crucial cell type in the first line of defense against the FD particles. When FD particles reach the lung, macrophages activate and phagocytosis occurs. This process is followed by an array of mechanisms including the release of inflammatory mediators such as chemokines and cytokines. Ultimately, NO production could lead to ROS production, which results in apoptosis [6]. This could result in various complications and among them, respiratory diseases, such as asthma and oxidative stress, due to ROS, rank above all. Therefore, mechanistic studies to stop or reduce this problem have gained attention among the scientific community. The present study focused on the activity of macrophage cells exposed to FD particles. It was evident that the FD particles could induce inflammation up to an optimum concentration of 125 μg mL^− 1^ without significantly affecting the viability of the macrophage cells. This suggests that NO production through the induction of FD could lead to the same results observed in previous studies. In order to reduce this effect, a green chemistry method was implemented, described as the SHE treatment.

*S. horneri*, a brown algae, has long been used as an edible seaweed [7] in the communities of the Asia-Pacific region. Its activity has been evaluated by many scientists. Heo et al. (2010) reported its potential anti-inflammatory effect under LPS-stimulated conditions in RAW macrophages. The compound of interest was fucoxanthine, which is a pigment dye found in brown algae [23]. Hoshino et al. (1998) reported on the active sulfated polysaccharides from *S. horneri* and its antiviral potential [24]. Its anti-inflammatory activity has also been evaluated numerous times for different extract types and partial purified chemicals such as ethanol extract and polysaccharide. Wen et al. (2016) discussed the anti-inflammatory effect of polysaccharides from *S. horneri* in RAW macrophages [25]. Sulfated polysaccharides from *S. horneri* have also been evaluated for their anti-oxidant and antitumor activities [26]. Kim et al. (2012) isolated chromene from *S. horneri* and evaluated its protective effect against the UV-A-induced damage in skin dermal fibroblasts [27]. However, to the best of our knowledge, this is the first report on “*S. horneri* ethanol extract” and its activity against FD-induced macrophage cells. *S. horneri* demonstrated effective anti-inflammatory activities against FD-induced macrophages. This effect was evaluated in inflammatory mediated pathways. It was evident that the effect was supported by the inhibition of pro inflammatory mediators such as NO, iNOS, COX-2, PGE_2_, and cytokines (TNF-α, IL-1β, and IL-6). Moreover, our results show that SHE suppresses the expression of the above-mentioned mRNA levels as well, leading to lower production of mediators required for inflammation, and hence inhibiting the inflammatory process. According to the results, in the FD-induced group, IL-6 and TNF-α showed significant upregulation compared to the LPS stimulation group. Hence, IL-6 and TNF-α could be used as biomarkers against FD identification in macrophage cells. NO production in the body is catalyzed by a family of enzymes named as nitric oxide synthases (NOSs). Among the many NOSs, iNOS is a vital isoform in the process, and it generates NO independently of intracellular calcium concentrations [28]. In cells, PGE_2_ is generated via arachidonic acid through catalysis of COX enzymes, and in this case, COX-2 appears to be the primary COX controlling PGE_2_ synthesis in response to inflammation. Multiple cytokines and growth factors induce COX-2 gene expression [29]. Thereby, controlling the cytokine levels could suppress the ultimate expression of NO through the mediated pathways. Immunological function multiplicity is regulated through intracellular regulatory proteins know as cytokines. They can induce prostaglandins and cause the production and release of acute-phase proteins [30].

As mentioned, because inflammation is induced by FD particles, the signaling array could cause possible ROS production, ultimately leading to cell death. This process could be identified as oxidative stress. In order to evaluate the SHE activity against FD-induced macrophages, the proteins and mRNA levels related to this pathway were also examined. The role of Nrf2/HO-1 in response to oxidative stress is considered an evolutionarily conserved mechanism. Nrf2 regulates the expression of genes coding for anti-oxidant, anti-inflammatory, and detoxifying proteins, and to some extent, the characteristics of Nrf2 are mimicked by its dependent genes (HO-1). HO-1 leads to production of iron ions, biliverdin, and CO, and among them, biliverdin readily transform to bilirubin. Bilirubin is a potent anti-oxidant. These products modulate inflammation and exhibit beneficial effects against oxidative stress [31]. Nrf2 in the cytosol is bound to a control protein known as Keap1 (Kelch-like ECH-associated protein 1). Under normal conditions, Keap1 promotes eventual degradation of Nrf2. However, under stressful conditions, Nrf2 is parted from Keap1, initiates translocation to the nucleus, and attaches to ARE (antioxidant response element) [32], initiating activation of transcription of cytoprotective genes such as HO-1. HO-1 is upregulated in the presence of nitric oxide, heavy metals, cytokines, etc. [33]. Hence, a dose-dependent upregulation of these two factors should be expected in the FD-induced macrophages treated with SHE. The results of the present study confirm that SHE acts as a potent protector against the effects of FD.

MAPKs phosphorylate specific target protein substrates, one of them being Nrf2/Keap1. These activations regulate a range of activities such as metabolism and programmed cell death. p38, ERK, and JNK are well-characterized sub families of MAPK, which only prevail in multicellular organisms. These could be used to control the activity of MAPK [34]. In this study, SB 202190 was used as a p38 inhibitor, which led to inhibition of the MAPK pathway signal transduction. Hence, this led to Nrf2 degradation in the cytoplasm, inhibiting the process of transcription of antioxidant genes. These observations suggest that SHE acts through the p38-activated MAPK-dependent Nrf2/HO-1 pathway.

## Conclusions

In conclusion, treatment with the compound SHE can reduce inflammation via downregulating the iNOS and COX-2 inflammatory mediators and also the pro-inflammatory cytokines in RAW 264.7 macrophages. This is supported by the mRNA expression levels of relevant mediators and cytokines. The inferred ROS production referred to as oxidative stress can also be relieved by the induction of HO-1 expression via the Nrf2 pathway by SHE treatment. This is suggested to play a key role in the protection of macrophage cells against FD particles. In addition, the results are supported by the possible p38 inhibition activity of SHE in the MAPK pathway and require more attention and further studies including gene expression analysis. The present study provides insight into the cytoprotective mechanism against FD particles. The treatment used here is low cost and considered a green chemistry method, which could have potential therapeutic use.

## Abbreviations

ARE: Antioxidant response element
COX-2: Cyclooxygenase-2
DMEM: Dulbecco’s modified Eagle’s medium
ELISA: Enzyme linked immunosorbent assay
FBS: Fetal bovine serum
FD: Fine dust
HO-1: Heme oxygenase 1
IL-1β: Interleukin 1 beta
IL-6: Interleukin 6
iNOS: Inducible Nitric oxide synthase
Keap1: Kelch-like ECH-associated protein 1
MAPK: Mitogen-activated protein kinase
MTT: 3-(4, 5-dimethylthiazol-2-yl)-2, 5-diphenyltetrazolium bromide
NO: Nitric oxide
Nrf2: Nuclear factor E2-related factor 2
PGE_2_: Prostaglandin E2
SHE: *Sargassum horneri* (Turner) C. Agardh ethanol extract
TNF-α: Tumor necrosis factor alpha
